# Lecture, Introductory to the Course on the Institutes of Medicine in the University of Pennsylvania

**Published:** 1852-02

**Authors:** 


ART. VII.—*Lecture, Introductory to the Course on the Institutes of Medicine in the University of Pennsylvaaia.* By SAMUEL JACKSON, M. D. Published by the Class. Philadelphia: 1851.

In this Lecture, Prof. Jackson asserts his clams to the authorship of many physiological doctrines now beginning to prevail. With a desire to do justice to the views he has maintained in his lectures for a number of years past, we present the following extracts from his late introductory, in which our readers will not fail to remark a striking resemblance to those of Dr. Carpenter quoted in our last number:

"The force presiding over the production of plastic matter is the same as the chemical force of organic bodies, and the primary and common organic form, a cell, may, as Mulder states, be the inevitable consequence of the special material. But whence come the special organic forms, so numerous, it may be said endless; so diversified and complex, yet always the same, and constructed on a plan perfect of its kind, and expressive of an ideal conception of the highest intelligence? Is it possible to regard the organic forms of living beings, bearing the impress of ideas that could originate only in the profoundest knowledge, and the very supremacy of wisdom, as proceeding from a force identical with chemical or physical forces, or any mere material force, or a necessary result of any material condition?

Employing the term force in its philosophical sense, as the expression of an unknown cause, I have been accustomed to ascribe this class of phenomena as exclusively belonging to organic force, or the radical force of life.

The special character of organic or radical force of life, if this view be correct, is modality, or the power of creating organic forms, the instruments and mechanisms of life. It possesses none of the attributes of the physical forces in its actions and influences. It has no identity with them; yet there is undoubted correlation.

Forms are immediately connected with matter, and organic forms with special protoplasms. These organizable materials are the product of chemical actions and play of chemical force, while this special direction of chemical force is depending on a definite temperature 98° to 100° in warm-blooded animals.

Again: organic matter is always the product of living beings; is prepared in organic or life instruments, under the chemical force which can accomplish those combinations only under the condition of a living organ endowed with organic force.

The correlation is evident. There exists a chain of sequences, each one of which is an indispensable link, necessary to the creation, in a material form, of an ideal type. But correlation and intimate dependency do not constitute identity of nature. A clear distinction is apparent between the force that prepares, by a chemical action, the material for constructing an organ or instrument, and the force that forms an ideal plan of the instrument or organ, and constructs it of this material.

Examples illustrating this proposition are furnished by the eye—an optical instrument; by the ear—an acoustic instrument; by the heart—an hydraulic engine; and the lungs, a pneumatic apparatus. Each of these is formed of numerous pieces, adjusted with the nicest precision, and adapted to perform a particular part necessary to the completion and working of the instrument. The material of which each separate piece is formed is special to that piece, yet has been derived from one common source, the blood, in which it did not exist. The organization or structure and the form are totally different in each, even at the point where they are in direct conjunction, and by no other structure or form could the especial organ or instrument be constructed. Complex results like these are not compatible with mere nutrition or growth.

The phenomena unquestionably the most characteristic of organic nature, are, the perpetual production of special organic matters, and repetitions, in successive generations, of the same typical forms, infinite in number and variety, expressed in the organization of living beings. Organic forms have continued through all time unchanged, invariable under the same circumstances, the offspring being the representatives of the parents.

These forms, evolved from a formless organic matter, were the first manifestations of the Divine Thought in the creation of the organic world. They emanated from the creative power of the Supreme Intelligence, moulding in material forms the ideal of the Eternal Mind."

